# Discordant Immune–Virologic Responses During Antiretroviral Therapy: Immune Dysregulation Patterns, CD4/CD8 Ratio Inversion, and Clinical Predictors in a Romanian HIV Cohort

**DOI:** 10.3390/v18050512

**Published:** 2026-04-29

**Authors:** Ruxandra-Cristina Marin, Radu Dumitru Moleriu, Gabriela S. Bungau, Delia Mirela Tit, Călin Muntean

**Affiliations:** 1 Doctoral School of Biological and Biomedical Sciences, University of Oradea, 410087 Oradea, Romania; marin.ruxandracristina@student.uoradea.ro (R.-C.M.); dtit@uoradea.ro (D.M.T.); 2 Discipline of Pharmacology, Clinical Pharmacology and Pharmacotherapy, “Carol Davila” University of Medicine and Pharmacy, 050474 Bucharest, Romania; 3 Department III, Functional Science, Discipline of Medical Informatics and Biostatistics, “Victor Babes”, University of Medicine and Pharmacy, 300041 Timisoara, Romania; cmuntean@umft.ro; 4 Department of Pharmacy, Faculty of Medicine and Pharmacy, University of Oradea, 410028 Oradea, Romania

**Keywords:** HIV, discordant response, immunological non-responder, virological discordance, CD4/CD8 ratio, immune dysregulation, opportunistic infections, antiretroviral therapy, Eastern Europe, Romania

## Abstract

(1) Background: Despite the success of combination antiretroviral therapy (cART), immune recovery in treated HIV infection remains heterogeneous, and discordant immune–virologic responses persist in a substantial proportion of people living with HIV (PLWH). These patterns may reflect ongoing immune dysregulation despite effective viral suppression. This study aimed to characterize discordant treatment classifications, evaluate immune imbalance using the CD4/CD8 ratio, identify associated clinical predictors, and assess opportunistic infection burden in a Romanian cohort of people living with HIV receiving long-term cART. (2) Methods: A retrospective cross-sectional study was conducted in 462 adults with HIV-1 infection receiving cART at the “Prof. Dr. Matei Balș” National Institute of Infectious Diseases, Bucharest (2018–2021). PLWH were classified as concordant responders (CR), immunological discordant responders (ID), or virological discordant responders (VD) based on plasma HIV-1 RNA and CD4+ T-cell count thresholds. Immune dysregulation was assessed using the CD4/CD8 ratio. Multinomial logistic, logistic, and negative binomial regression models were used to identify predictors of discordant responses, severe CD4/CD8 ratio inversion, and opportunistic infection burden. (3) Results: Discordant responses were observed in 30.7% of PLWH (14.5% ID, 16.2% VD). CD4/CD8 ratio inversion occurred in 71.2% and severe inversion in 40.0%. Significant differences across clinical classification groups were found for CD4+T-cell counts (H = 153.62, *p* < 0.001, ε^2^ = 0.33) and CD4/CD8 ratio (H = 115.10, *p* < 0.001, ε^2^ = 0.25), while CD8+ counts were similar (*p* = 0.571). Male sex was associated with both ID and VD, and severe CD4/CD8 inversion was strongly associated with ID. Opportunistic infection burden was associated with duration of HIV infection and CDC stage. (4) Conclusions: Discordant immune–virologic responses remain frequent during long-term cART and are characterized by persistent immune imbalance reflected by CD4/CD8 ratio inversion. The CD4/CD8 ratio may provide clinically relevant information on immune recovery beyond CD4+ T-cell counts.

## 1. Introduction

Human immunodeficiency virus (HIV) infection has evolved from a uniformly fatal disease into a manageable chronic condition following the introduction and widespread implementation of combination antiretroviral therapy (cART). Contemporary antiretroviral regimens effectively suppress viral replication, facilitate immune reconstitution, and substantially improve life expectancy among people living with HIV (PLWH) [1].

The primary goals of cART include sustained suppression of plasma HIV-1 RNA to undetectable levels, restoration and preservation of CD4+ T-lymphocyte counts, reduction in HIV-related morbidity and mortality, and improvement of long-term quality of life. The global scale-up of antiretroviral therapy has profoundly transformed HIV care over the past two decades. According to recent global estimates, approximately 77% of people living with HIV worldwide were receiving antiretroviral therapy in 2023–2024, and about 73% had achieved viral suppression, reflecting substantial progress toward the UNAIDS 95-95-95 targets and highlighting the impact of large-scale treatment programs on HIV outcomes [2].

Despite the high efficacy of modern cART, immune restoration following virological suppression remains heterogeneous. A considerable proportion of treated individuals fail to achieve concordant immunological and virological responses, a phenomenon broadly referred to as a discordant treatment response. Immunological discordance, often termed immunological non-response, is characterized by persistently low CD4+ T-cell counts despite sustained viral suppression, whereas virological discordance refers to recovery of CD4+ T-cell counts despite detectable or intermittent viremia. Immunological non-response remains a significant clinical concern, affecting approximately 20–30% of individuals receiving long-term ART [3]. These discordant response patterns have been consistently associated with increased risks of both AIDS-defining and non-AIDS-related clinical events, including cardiovascular disease, malignancies, chronic kidney disease, and overall mortality [4].

Incomplete immune recovery is increasingly recognized as a complex immunopathological state rather than simply a quantitative failure of CD4+ T-cell restoration. Persistent immune dysregulation during treated HIV infection is characterized by chronic systemic inflammation, sustained immune activation, T-cell senescence, and functional exhaustion of both innate and adaptive immune compartments. Immunological non-responders demonstrate persistent T-cell activation and impaired cytokine responses despite effective viral suppression, indicating the persistence of qualitative immune defects [5,6]. From a pathophysiological perspective, immunological non-response reflects a state of persistent immune activation and chronic inflammation rather than solely a deficit in CD4+ T-cell expansion [7].

Several mechanisms may sustain immune dysfunction even during suppressive ART. Residual antigenic stimulation, dysregulated innate immune signaling, and inflammatory feedback loops may impair lymphocyte homeostasis and tissue repair [8]. In addition, disruption of gut epithelial integrity and microbial translocation remain central components of HIV immunopathology. Translocated microbial products can maintain monocyte and macrophage activation and contribute to systemic inflammation, thereby counteracting immune reconstitution despite controlled plasma viremia [9].

Among the immunological markers proposed to evaluate immune recovery during ART, the CD4/CD8 ratio has emerged as a particularly informative biomarker. In healthy individuals, this ratio typically ranges from 1.0 to 4.0; however, HIV infection commonly results in persistent ratio inversion due to both CD4+ T-cell depletion and chronic expansion of activated CD8+ T cells [10,11]. Normalization of this ratio is not consistently achieved even after successful cART. A persistently low CD4/CD8 ratio has been associated with ongoing immune activation, immunosenescence, and an increased risk of serious non-AIDS clinical events, including cardiovascular disease and malignancies, independent of absolute CD4+T-cell counts [12,13].

Multiple processes may contribute to impaired immune reconstitution, including reduced thymic output and diminished naïve T-cell production, persistent immune activation driven by residual viral replication or antigenic stimulation, and disruption of the gut mucosal barrier leading to microbial translocation and systemic inflammation [14]. Chronic immune activation may also promote the upregulation of inhibitory checkpoint receptors on T cells, such as programmed cell death protein-1 (PD-1), contributing to T-cell exhaustion and functional impairment. In addition to these immunological mechanisms, host-related factors may influence immune recovery trajectories. Advanced age, delayed initiation of antiretroviral therapy, and a low CD4+ T-cell nadir prior to treatment have all been associated with poorer immune reconstitution. Coinfections, especially hepatitis B virus (HBV) and hepatitis C virus (HCV), as well as genetic and metabolic host factors, may further amplify immune activation and accelerate immune senescence, thereby impairing restoration of immune homeostasis [15,16].

Importantly, individuals with incomplete immune recovery remain at increased risk for opportunistic infections and chronic comorbidities even when viral suppression is achieved. Studies have demonstrated that immunological non-responders experience higher rates of AIDS-defining illnesses, including pneumocystis pneumonia and opportunistic fungal infections, as well as an increased incidence of non-AIDS-related diseases such as cardiovascular disorders and malignancies [4].

Virological discordance (VD) and intermittent low-level viremia (LLV) have also been increasingly recognized as clinically relevant phenomena during antiretroviral therapy and may contribute to incomplete immune recovery despite otherwise effective treatment. In a large longitudinal cohort including 7485 individuals followed through 2021, LLV trajectories between 50 and 999 copies/mL were observed in approximately 9.8% of people living with HIV (PLWH) and were independently associated with a reduced probability of CD4+ T-cell recovery compared with individuals maintaining sustained viral suppression below 50 copies/mL [17].

Persistent LLV during cART has also been associated with an increased risk of subsequent virological failure. Factors such as incomplete adherence, pharmacokinetic variability, drug–drug interactions, and the emergence of minority resistant viral variants may contribute to intermittent viral replication despite ongoing treatment. Accordingly, detectable viremia during therapy is considered clinically relevant even when viral loads remain below conventional failure thresholds [18,19]. Also, intermittent LLV may originate from long-lived HIV reservoirs and clonally expanded infected CD4+ T cells capable of sporadic viral expression despite suppressive therapy, thereby sustaining chronic antigenic stimulation and immune activation [20].

Different patterns of detectable viremia during ART should also be distinguished. A viral blip refers to a single transient episode of detectable HIV RNA followed by a return to suppression, whereas low-level viremia describes persistent viral loads above the limit of detection but below conventional thresholds for virologic failure, typically between 50 and 200 copies/mL. Virologic failure, in contrast, is defined as persistent HIV RNA levels ≥200 copies/mL despite ongoing therapy. While isolated viral blips are generally considered clinically benign, persistent LLV has been associated with viral evolution, drug resistance, and an increased risk of virologic failure, emphasizing the importance of careful monitoring of viral load dynamics in treated individuals [21,22].

The HIV epidemic in Romania presents distinctive epidemiological characteristics compared with those observed in Western Europe or sub-Saharan Africa. A substantial proportion of the current adult PLWH originates from a cohort infected during early childhood through parenteral exposure in the late 1980s and early 1990s. This group represents one of the largest long-term survivor cohorts of pediatric HIV infection worldwide and is characterized by prolonged infection duration, complex treatment histories, and cumulative exposure to multiple antiretroviral regimens. These factors may influence immune recovery patterns and long-term clinical outcomes [23].

Furthermore, coinfections with hepatotropic viruses remain relatively common in the Romanian HIV population, particularly hepatitis B virus (HBV) and hepatitis C virus (HCV). These coinfections may contribute to persistent immune activation, altered immune cell homeostasis, and delayed immune recovery during antiretroviral therapy. Despite these unique epidemiological and clinical characteristics, data regarding discordant immune–virologic responses in Eastern European HIV cohorts remain limited [24].

Therefore, given that discordant immune–virologic responses remain insufficiently characterized in Eastern European HIV cohorts, especially in populations with long-standing infection and complex treatment histories such as the Romanian cohort, the present study aimed to characterize patterns of immune dysregulation and discordant immunologic and virologic responses among people living with HIV receiving long-term antiretroviral therapy. Specifically, we sought to determine the prevalence and immunologic profiles of concordant, immunologically discordant, and virologically discordant treatment responses; to quantify the extent of persistent CD4/CD8 ratio inversion as an indicator of residual immune dysregulation; to identify clinical, immunologic, and therapeutic factors associated with these discordant treatment responses; to assess the impact of discordance on the cumulative burden and spectrum of opportunistic infections; and to evaluate the CD4/CD8 ratio as a marker of incomplete immune reconstitution.

## 2. Materials and Methods

### 2.1. Study Design and Population

This retrospective cross-sectional study was conducted using clinical and laboratory data from adult PLWH with confirmed HIV-1 infection receiving combination antiretroviral therapy (cART) at the “Prof. Dr. Matei Balș” National Institute of Infectious Diseases in Bucharest, Romania. The analysis included PLWH evaluated between October 2018 and December 2021. Data collection and analysis were conducted retrospectively following approval by the institutional Ethics Committee.

A total of 462 individuals were included in the study. Eligible participants were required to be at least 18 years of age, to have confirmed HIV-1 infection, and to have received any cART regimen for a minimum of six months prior to evaluation in order to allow stabilization of virological and immunological responses to therapy. PLWH with incomplete clinical or laboratory records for key study variables were excluded from the analysis.

Demographic, clinical, laboratory, and treatment-related information was extracted from the electronic medical records of the Institute. The collected data included age at the time of evaluation, sex, and environment of residence (urban or rural). Clinical variables included age at HIV diagnosis, duration of HIV infection, CDC clinical stage, and documented history of opportunistic infections. The duration of HIV infection was calculated as the time elapsed from the documented date of HIV diagnosis to the date of the most recent clinical evaluation. Laboratory parameters comprised CD4+ and CD8+ T-cell counts, CD4/CD8 ratio, plasma HIV-1 RNA viral load, and CD4 nadir. Information regarding antiretroviral treatment included the duration of therapy, the number of prior antiretroviral regimens, antiretroviral drug classes used, and overall treatment burden.

All clinical and laboratory parameters were recorded from the most recent routine clinical evaluation available during the study period.

### 2.2. Definitions and Treatment Response Clinical Classification

Treatment response categories were defined using the most recent available CD4+ T-cell count and plasma HIV-1 RNA viral load measurements. PLWH were classified into concordant or discordant response groups according to the relationship between virological suppression and immunological recovery. To ensure conceptual clarity and reduce potential misclassification bias, these operational definitions were selected based on clinically established thresholds and prior literature, while acknowledging the heterogeneity of definitions used across studies evaluating discordant immune–virologic responses [12].

These definitions are consistent with clinically established thresholds and widely used classifications in studies evaluating discordant immune–virologic responses during antiretroviral therapy, facilitating comparability with other cohorts [25,26].

Treatment response group stratification was operationally defined based on the most recent available measurements of plasma HIV-1 RNA and CD4+ T-cell counts. A concordant response (CR) was considered when virological suppression and immunological recovery were both present, defined as plasma HIV-1 RNA levels below 50 copies/mL together with a CD4+ T-cell count greater than 200 cells/µL. Immunological discordant response (ID) was defined as persistent immunological impairment despite virological suppression, corresponding to HIV-1 RNA levels below 50 copies/mL (standard threshold used to define virological suppression in clinical monitoring of antiretroviral therapy) in combination with a CD4+ T-cell count of 200 cells/µL or lower. Virological discordant response (VD) was defined as detectable viremia in the presence of preserved immunological status, corresponding to HIV-1 RNA ≥50 copies/mL in the presence of CD4+ T-cell counts >200 cells/µL. To account for biological heterogeneity within this category, individuals were further stratified into low-level viremia (50–199 copies/mL) and virological failure (≥200 copies/mL), in accordance with current clinical guidelines [21]. For descriptive purposes, virological failure was additionally stratified into moderate-level viremia (200–999 copies/mL) and high-level viremia (≥1000 copies/mL) to better characterize the distribution of viral replication within this group.

The CD4 threshold of 200 cells/µL was selected because it represents a clinically established marker of severe immunosuppression and has been widely used in previous studies evaluating discordant immune responses during antiretroviral therapy.

Additional operational definitions were applied for virological and immunological parameters. Virological suppression was defined as plasma HIV-1 RNA levels below 50 copies/mL. Low-level viremia was defined as persistent plasma HIV-1 RNA levels above the lower limit of detection of the assay but below 200 copies/mL. Virological failure was defined as persistent plasma HIV-1 RNA levels ≥ 200 copies/mL despite ongoing antiretroviral therapy.

Immune dysregulation was further assessed using the CD4/CD8 ratio. Ratio inversion was defined as values below 1.0, severe inversion as values of 0.50 or lower, and ratio normalization as values equal to or above 1.0. The CD4/CD8 ratio has been increasingly recognized as an indicator of immune imbalance and persistent immune activation in individuals receiving antiretroviral therapy [21,27,28].

### 2.3. Opportunistic Infections and Clinical Classification

Opportunistic infections were assessed based on the documented cumulative clinical history recorded in the individuals’ medical records. Information regarding the presence of opportunistic infections was retrospectively extracted from the medical files and electronic clinical records, and included diagnoses previously established during routine clinical care and documented by the treating physicians. No additional diagnostic investigations were performed for the purpose of this study. The conditions considered in the analysis included cytomegalovirus infection, herpes simplex virus infection, Epstein–Barr virus infection, tuberculosis, candidiasis, *Mycoplasma pneumoniae* infection, toxoplasmosis, salmonellosis, *Pneumocystis jirovecii* pneumonia, and Kaposi sarcoma.

Clinical staging of HIV infection was classified according to the 1993 Centers for Disease Control and Prevention (CDC) revised surveillance case definition for HIV infection, which incorporates clinical conditions and CD4+ T-cell count categories to stratify disease severity [28].

### 2.4. Laboratory Measurements

Immunological parameters were determined using multiparameter flow cytometry. Absolute CD4+ and CD8+ T-lymphocyte counts were measured using the BD FACSCalibur™ flow cytometry platform (Becton Dickinson, San Jose, CA, USA) and expressed as cells per microliter of peripheral blood. The CD4/CD8 ratio was calculated directly from the absolute counts obtained during the same analysis.

Plasma HIV-1 RNA viral load was quantified using a standardized real-time reverse transcription polymerase chain reaction assay (Abbott RealTime HIV-1, Abbott Molecular, Des Plaines, IL, USA), which has a lower limit of detection of 50 copies/mL. Virological monitoring is routinely used to assess the effectiveness of antiretroviral therapy and detect virological failure during long-term treatment.

Information regarding viral hepatitis coinfections was obtained retrospectively from the individuals’ medical records and laboratory reports available in the electronic clinical database of the institute. The hepatitis B and hepatitis C status reflected serological testing previously performed during routine clinical care in the central laboratory of the institute. Hepatitis B virus infection was determined by the detection of hepatitis B surface antigen (HBsAg), while hepatitis C virus infection was assessed by the presence of anti-HCV antibodies using automated immunoassay platforms. No additional laboratory testing was performed for the purposes of this study.

### 2.5. Statistical Analysis

All statistical analyses were conducted using JASP (v0.96) and RStudio (version 4.3.2), integrating the R statistical computing environment for advanced data processing and modeling. Descriptive and inferential statistical procedures were implemented through the analytical modules available in JASP and corresponding R packages. Graphical representations and exploratory data visualizations were generated within RStudio, utilizing the ColorBrewer “Paired” qualitative palette to ensure perceptually distinct and colorblind-friendly categorical encoding.

Continuous variables were summarized as median and interquartile range (IQR), given non-normal distributions verified by the Shapiro–Wilk test. Categorical variables were reported as absolute frequencies and proportions. Prevalence estimates were accompanied by 95% Wilson score confidence intervals.

Comparisons of continuous variables across the three phenotypic groups were performed using the Kruskal–Wallis H test, with post hoc pairwise Mann–Whitney U tests and Bonferroni correction. Effect sizes were reported as epsilon-squared (ε^2^). Associations between categorical variables were assessed using Pearson’s chi-squared test with Cramér’s V as the effect size.

Multinomial logistic regression was employed to identify determinants of discordant responses, with CR as the reference category. Duration of ART was used instead of duration of HIV infection due to high collinearity (Spearman ρ = 0.809). Results were expressed as relative risk ratios (RRR) with 95% confidence intervals. Binary logistic regression was used to identify predictors of severe CD4/CD8 ratio inversion (≤0.5), with model performance assessed by the area under the receiver operating characteristic curve (AUC), McFadden’s pseudo-R^2^, and Hosmer–Lemeshow goodness-of-fit. For opportunistic infection models, both binary logistic regression (any OI) and negative binomial regression (OI count, to account for overdispersion) were fitted with identical predictor sets.

Bivariate correlations between the CD4/CD8 ratio and continuous clinical variables were assessed using Spearman’s rank correlation coefficient with Benjamini–Hochberg false discovery rate correction. All tests were two-sided with α = 0.05. In accordance with current recommendations, emphasis was placed on effect size estimates and confidence intervals rather than exclusive reliance on *p*-value thresholds [29].

### 2.6. Ethical Considerations

The study was conducted in accordance with the principles of the Declaration of Helsinki and was approved by the Ethics Committee of the “Prof. Dr. Matei Balș” National Institute of Infectious Diseases (approval no. C05865/4 May 2021). Given the retrospective design of the study and the use of anonymized data extracted from medical records, the requirement for individual informed consent was waived. All patient data were processed in a de-identified format to ensure confidentiality and data protection.

## 3. Results

### 3.1. Cohort Characteristics and Distribution of Treatment Response Clinical Classification

The study cohort included 462 individuals living with HIV who were receiving long-term antiretroviral therapy. The median age was 40 years (IQR 35–50), and 61.9% were male. Participants had a long history of infection and treatment exposure, with a median duration of HIV infection of 18 years (IQR 11–25) (calculated from the time of diagnosis) and a median duration of cART of 14 years (IQR 9.0–21.8). A substantial proportion of PLWH had experienced advanced disease during their clinical course, with 62.8% classified as CDC stage C. The median CD4+ nadir was 163 cells/µL (IQR 53–314), and PLWH had received a median of five previous antiretroviral regimens (IQR 2–12). Coinfections were frequent, affecting 37.2% of individuals with hepatitis C virus and 41.1% with hepatitis B virus.

At the time of assessment, the median CD4+ T-cell count was 532.5 cells/µL (IQR 309–765), the median CD8+ T-cell count was 788 cells/µL (IQR 565–1067), and the median CD4/CD8 ratio was 0.6 (IQR 0.4–1.1). Viral suppression (<50 copies/mL) was achieved in 83.8% of PLWH, while 7.7% had low-level viremia (50–199 copies/mL) and 8.5% met criteria for virological failure (≥200 copies/mL).

Participants were subsequently categorized into three treatment response groups based on clinical classification: concordant response (CR), immunological discordance (ID), and virological discordance (VD). Concordant response was defined as viral suppression (<50 copies/mL) with CD4+ T-cell count >200 cells/µL, immunological discordance as viral suppression with CD4+ T-cell count ≤200 cells/µL, and virological discordance as detectable viremia (≥50 copies/mL) in the presence of CD4+ T-cell count >200 cells/µL.

Within the virological discordance group, detectable viremia was heterogeneous. Specifically, 36 individuals (48.0%) had low-level viremia (50–199 copies/mL), while 39 individuals (52.0%) had virological failure (≥200 copies/mL). Among those with virological failure, 10 individuals (13.3%) had viral loads between 200 and 999 copies/mL, and 29 individuals (38.7%) had viral loads ≥1000 copies/mL. These findings indicate substantial variability in virological profiles within this group.

The distribution of demographic, clinical, immunological, and virological characteristics across these group stratifications is summarized in Table 1. Concordant immune–virologic responses were observed in 320 PLWH (69.3%, 95% CI 64.9–73.3%), whereas discordant responses were identified in 142 individuals (30.7%, 95% CI 26.7–35.1%). Among the discordant cases, 67 individuals (14.5%, 95% CI 11.6–18.0%) presented immunological discordance and 75 individuals (16.2%, 95% CI 13.2–19.9%) presented virological discordance. Overall, CD4/CD8 ratio inversion (<1.0) was observed in 71.2% of PLWH, including severe inversion (≤0.5) in 40.0%. The prevalence of ratio inversion varied across treatment response classification, with the highest proportion observed among individuals with immunological discordance.

Descriptive statistical analysis was performed to further characterize the distribution of plasma HIV-1 RNA levels within the virological discordance group (Table 2). Given the skewed distribution of viral load values, the median was used as a measure of central tendency, while variability was described using the IQR. Minimum and maximum values were additionally reported to capture the full range of observed values. The analysis revealed substantial heterogeneity in plasma viral load within this group, with values spanning from low-level viremia to high-level virological failure.

### 3.2. Immune Parameters Across Treatment Response Clinical Classification Groups

Significant differences were observed in both the CD4/CD8 ratio (Kruskal–Wallis H = 115.10, *p* < 0.001, ε^2^ = 0.25) and CD4+ T-cell count (H = 153.62, *p* < 0.001, ε^2^ = 0.33). The CD4/CD8 ratio was lowest in the immunological discordance group (median 0.2, IQR 0.2–0.3), compared with concordant responders (0.8, IQR 0.5–1.2) and individuals with virological discordance (0.6, IQR 0.4–0.8). Pairwise comparisons confirmed significant differences between all clinical classification groups after Bonferroni correction (all *p* adj < 0.01).

CD8+ T-cell counts did not differ significantly across the three groups (H = 1.12, *p* = 0.571, ε^2^ = 0.002), with median values of 790.5 cells/µL in the concordant response group, 755 cells/µL in the immunological discordance group, and 792 cells/µL in the virological discordance group.

**Figure 1:**
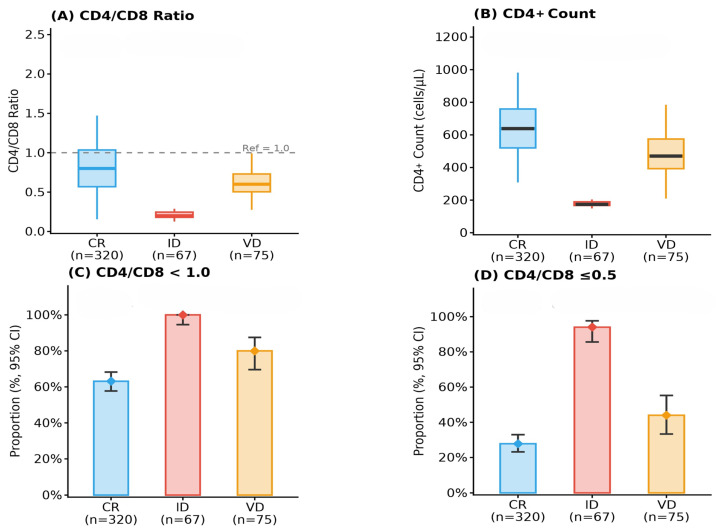
Immune parameters across treatment response clinical classification groups. (**A**) Distribution of CD4/CD8 ratio by clinical classification (Kruskal–Wallis test). (**B**) Distribution of CD4+ T-cell counts across treatment response clinical classification. (**C**) Prevalence of CD4/CD8 ratio inversion (<1.0) across response clinical classification. (**D**) Prevalence of severe CD4/CD8 ratio inversion (≤0.5) across response clinical classification. CR, concordant response; ID, immunological discordance; VD, virological discordance.

The distribution of CD4/CD8 ratio categories also differed significantly across treatment response classification. Ratio inversion (<1.0) was present in all PLWH with immunological discordance and in a majority of those with virological discordance and concordant responses (χ^2^ = 48.12, *p* < 0.001, Cramér’s V = 0.295). Severe inversion (≤0.5) was most frequent among individuals with immunological discordance (94.0%), compared with 44.0% in virological discordance and 27.8% in concordant responders (χ^2^ = 101.76, *p* < 0.001, Cramér’s V = 0.469). Comparisons of immune markers across treatment response groups’ stratification are summarized in Table 3 and for visual comparison, the immune parameters showing statistically significant differences across clinical classification are graphically displayed in Figure 1.

### 3.3. Determinants of Discordant Treatment Responses

**Figure 2:**
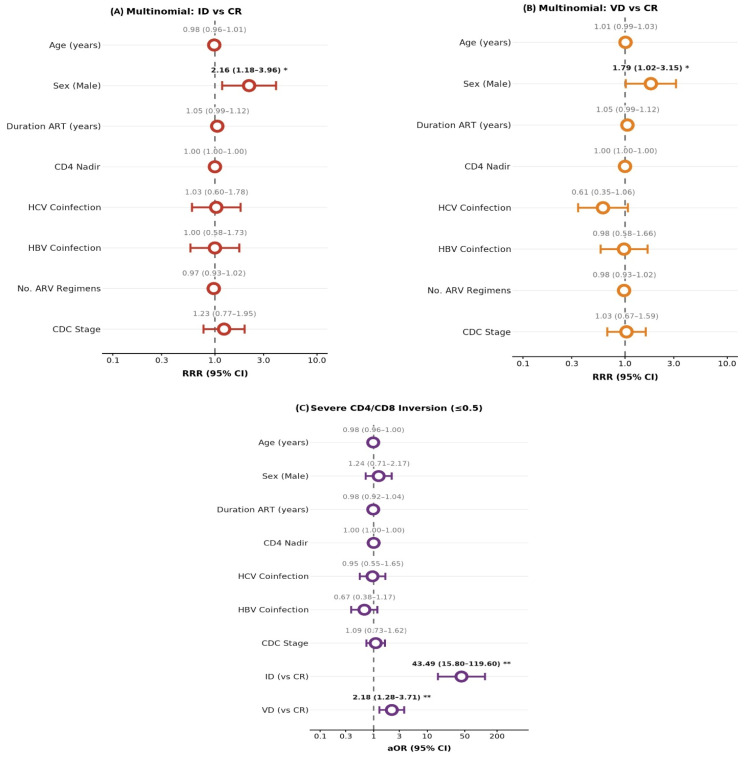
Regression analyses of factors associated with discordant treatment responses and severe CD4/CD8 ratio inversion. (**A**) Multinomial logistic regression analysis identifying predictors of ID compared with CR; (**B**) Multinomial logistic regression analysis identifying predictors of VD compared with CR. (**C**) Binary logistic regression analysis evaluating predictors of severe CD4/CD8 ratio inversion (≤0.5). CR, concordant response; ID, immunological discordance; VD, virological discordance; ART, antiretroviral therapy; HCV, hepatitis C virus; HBV, hepatitis B virus; CDC, Centers for Disease Control and Prevention. The vertical dashed line represents the null value (RRR or aOR = 1). Error bars indicate 95% confidence intervals. Asterisks denote statistical significance (*, *p* < 0.05; **, *p* < 0.01).

A multinomial logistic regression model was used to evaluate clinical predictors of immunological discordance (ID) and virological discordance (VD) relative to concordant response (CR). The complete regression results are presented in Table 4**,** while the effect estimates and their confidence intervals are graphically illustrated in Figure 2A,B, with statistically significant associations highlighted. The overall model did not reach statistical significance (likelihood ratio χ^2^ = 20.78, *p* = 0.187; pseudo-R^2^ = 0.027).

Male sex was significantly associated with both discordant treatment responses. The relative risk ratio (RRR) for immunological discordance was 2.16 (95% CI 1.18–3.96, *p* = 0.013), and for virological discordance, 1.79 (95% CI 1.02–3.15, *p* = 0.044), as illustrated in Figure 2A,B.

Duration of antiretroviral therapy showed a positive but non-significant association with both ID (RRR = 1.05, *p* = 0.093) and VD (RRR = 1.05, *p* = 0.078). A non-significant inverse association was observed between HCV coinfection and VD (RRR = 0.61, 95% CI 0.35–1.07, *p* = 0.082). Age, CD4 nadir, number of prior antiretroviral regimens, HBV coinfection, and CDC clinical stage were not significantly associated with either discordant treatment response.

In addition, the association between treatment response groups’ stratification and severe CD4/CD8 ratio inversion (≤0.5) was evaluated using binary logistic regression, and the adjusted effect estimates are shown in Figure 2C. In this analysis, immunological discordance showed a strong association with severe ratio inversion, while virological discordance demonstrated a more moderate association compared with concordant responders.

### 3.4. Predictors of Severe CD4/CD8 Ratio Inversion

**Figure 3:**
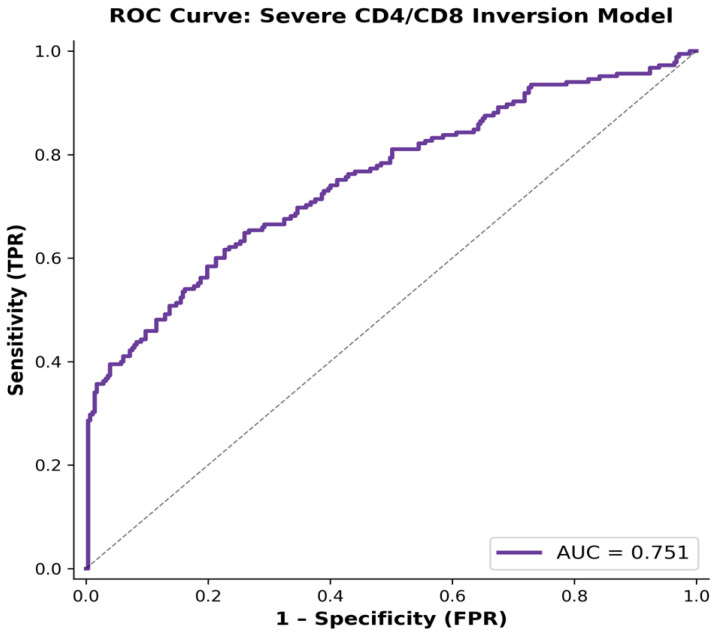
Receiver operating characteristic (ROC) curve for the model predicting severe CD4/CD8 ratio inversion (≤0.5). The model demonstrated moderate discriminative ability with an area under the curve (AUC) of 0.751.

Binary logistic regression was performed to identify factors associated with severe CD4/CD8 ratio inversion (≤0.5). The results are presented in Table 5, and the model discrimination is illustrated in Figure 3. The model demonstrated moderate discriminative performance, with an area under the ROC curve (AUC) of 0.751 and a McFadden pseudo-R^2^ of 0.194.

Treatment response classification was the strongest predictor of severe ratio inversion. Compared with concordant responders, immunological discordance was associated with markedly higher odds of severe inversion (adjusted odds ratio [aOR] = 43.49, 95% CI 15.17–124.67, *p* < 0.001). Virological discordance was also associated with increased odds of severe inversion (aOR = 2.18, 95% CI 1.28–3.72, *p* = 0.004).

None of the other variables included in the model reached statistical significance. Age (aOR = 0.98, *p* = 0.075) and HBV coinfection (aOR = 0.67, *p* = 0.085) showed non-significant trends, while sex, duration of ART, CD4+ nadir, HCV coinfection, and CDC clinical stage were not associated with severe inversion.

### 3.5. Correlation Structure of CD4/CD8 Ratio Determinants

**Figure 4:**
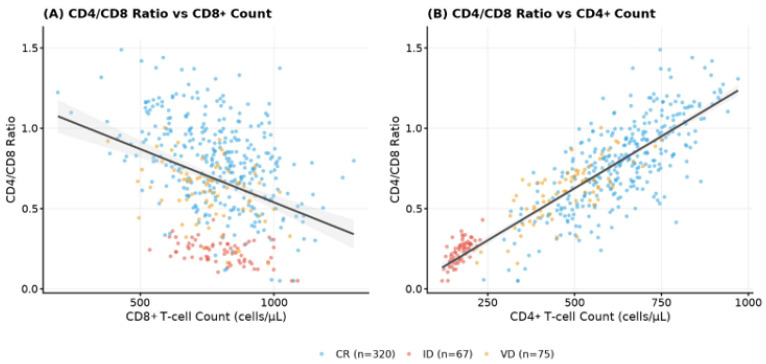
Correlation between CD4/CD8 ratio and immune cell counts. Scatter plots showing the relationship between CD4/CD8 ratio and (**A**) CD8+ T-cell count and (**B**) CD4+ T-cell count. Points are color-coded according to treatment response clinical classification (CR, concordant response; ID, immunological discordance; VD, virological discordance).

Spearman correlation analyses were performed to evaluate associations between the CD4/CD8 ratio and selected clinical variables (Table 6). Significant correlations were observed with both CD4+ and CD8+ T-cell counts. The CD4/CD8 ratio showed a strong positive correlation with CD4+ count (ρ = 0.791, *p* < 0.001) and a moderate inverse correlation with CD8+ count (ρ = −0.563, *p* < 0.001) (Figure 4). Both associations remained significant after false discovery rate (FDR) correction.

No significant correlations were observed between the CD4/CD8 ratio and CD4+ nadir (ρ = −0.069, *p* = 0.138), age (ρ = 0.065, *p* = 0.165), duration of HIV infection (ρ = 0.000, *p* = 0.999), or duration of ART (ρ = −0.015, *p* = 0.754).

### 3.6. Viral Hepatitis Coinfection

The prevalence of viral hepatitis coinfections did not differ significantly across treatment response groups (Table 7). HCV coinfection was present in 38.8% of concordant responders, 40.3% of PLWH with immunological discordance, and 28.0% of those with virological discordance (χ^2^ = 3.32, *p* = 0.190, Cramér’s V = 0.085). HBV coinfection showed a similar distribution across groups (CR 40.6%, ID 43.3%, VD 41.3%; χ^2^ = 0.16, *p* = 0.922, Cramér’s V = 0.019).

### 3.7. Opportunistic Infection History

A history of at least one opportunistic infection (OIs) was reported in 236 individuals (51.1%), with no significant differences across treatment response groups (χ^2^ = 0.12, *p* = 0.940, Cramér’s V = 0.016). Associations between clinical variables and OI history were evaluated using binary logistic regression, while predictors of the number of OIs were examined using a negative binomial regression model (Table 8).

In the binary logistic model, duration of HIV infection was the only variable significantly associated with the presence of any OI (adjusted odds ratio [aOR] = 0.964, 95% CI 0.939–0.990, *p* = 0.007). In the negative binomial model, both duration of HIV infection (incidence rate ratio [IRR] = 0.977, 95% CI 0.963–0.990, *p* < 0.001) and CDC clinical stage (IRR = 1.192, 95% CI 1.010–1.406, *p* = 0.037) were significantly associated with the number of OIs.

Treatment response classification was not associated with OI history or OI count in either model.

## 4. Discussion

In this cross-sectional study of a long-term treated Romanian HIV cohort, discordant immune–virologic responses remained relatively common, affecting nearly one-third of PLWH despite prolonged antiretroviral therapy. This proportion is consistent with observations from contemporary international cohorts, where incomplete or discordant immune recovery persists in approximately 15–30% of individuals receiving suppressive antiretroviral therapy [3,30].

These findings indicate that although modern combination antiretroviral therapy effectively suppresses viral replication in most treated individuals, restoration of immune homeostasis remains heterogeneous. The persistence of discordant immune–virologic patterns reflects the complex interaction between virological suppression, immune reconstitution, and chronic immune dysregulation during treated HIV infection [8].

The distribution of discordant treatment responses observed in our cohort (14.5% immunological discordance and 16.2% virological discordance) may partly reflect the distinctive epidemiological characteristics of the Romanian HIV epidemic. A substantial proportion of individuals belong to the cohort infected during early childhood in the late 1980s and early 1990s, resulting in long infection duration and extensive cumulative exposure to antiretroviral therapy. These individuals represent long-term survivors of pediatric HIV infection and frequently present complex treatment histories and advanced historical disease stages, factors that may contribute to heterogeneous immune recovery during long-term therapy [31]. Several studies have demonstrated that immunological status at the time of diagnosis strongly influences long-term recovery trajectories. Individuals presenting with advanced immunosuppression often experience slower or incomplete CD4+ T-cell restoration despite sustained virological suppression during treatment [27,32,33]. Importantly, the virological discordance group included individuals with both low-level viremia and viral loads meeting the threshold for virological failure, which represent biologically distinct scenarios. However, given the cross-sectional design and the use of a single viral load measurement, this classification does not necessarily reflect confirmed or persistent virological failure as defined in longitudinal clinical monitoring. Rather, it encompasses a spectrum of virological profiles, ranging from transient or low-level viral replication to potentially sustained viremia. Although these conditions share the feature of detectable viremia in the presence of preserved CD4+ T-cell counts, they may reflect different underlying mechanisms, including adherence variability, pharmacokinetic factors, or the possible emergence of drug resistance. Accordingly, virological discordance should be interpreted as a heterogeneous clinical category rather than a single biological entity. This distinction is particularly relevant in the absence of longitudinal viral load data and drug resistance testing.

An important observation in our study is the stability of CD8+ T-cell counts across treatment response group stratification. Despite clear differences in CD4+ counts and CD4/CD8 ratios between concordant responders and individuals with discordant responses, CD8 levels remained consistently elevated across all groups. Although a local HIV-negative reference range was not available within our dataset, CD8+ T-cell counts in healthy individuals are typically reported within a range of approximately 300–900 cells/µL [25], whereas the median value observed in our cohort (790 cells/µL) lies toward the upper end of this range, supporting the presence of persistent immune activation. This suggests that persistent CD8+ expansion represents a common immunological background in long-term treated HIV infection rather than a feature specific to a given clinical classification. Sustained expansion of activated and differentiated CD8+ T cells has been widely documented in individuals receiving suppressive antiretroviral therapy and is considered a hallmark of persistent immune activation [34,35].

Persistent CD8+ count expansion is generally attributed to chronic immune stimulation during treated HIV infection. Residual viral antigen expression from long-lived reservoirs, microbial translocation due to disruption of gut mucosal integrity, and chronic coinfections, especially cytomegalovirus, have all been implicated in maintaining immune activation despite effective viral suppression. These processes sustain inflammatory signaling and drive differentiation of cytotoxic T-cell populations during long-term therapy [36,37,38].

Within this context, variation in the CD4/CD8 ratio appears primarily determined by the degree of CD4+ T-cell recovery rather than major changes in the CD8+ compartment itself. Our correlation analyses support this interpretation, demonstrating a strong positive association between the CD4/CD8 ratio and CD4+ count, together with a moderate inverse correlation with CD8+ count. Similar patterns have been reported in other treated cohorts, where persistent CD8+ elevation contributes to long-term ratio inversion and reflects residual immune dysfunction during suppressive therapy [33].

Beyond reflecting the numerical balance between T-cell subsets, the CD4/CD8 ratio is increasingly regarded as an integrative indicator of immune restoration during antiretroviral therapy. Persistent ratio inversion reflects the combined effects of incomplete CD4+ recovery, chronic CD8+ activation, and sustained inflammatory signaling, processes that collectively characterize incomplete immune reconstitution. Several studies therefore propose the CD4/CD8 ratio as a clinically informative marker of long-term immune health in people living with HIV receiving suppressive therapy [39,40,41].

Importantly, CD4/CD8 ratio inversion remained highly prevalent even among concordant responders, affecting more than 60% of PLWH in this group. This finding indicates that viral suppression and recovery of CD4+ counts above clinical thresholds do not necessarily correspond to full normalization of immune homeostasis. Persistent immune alterations during suppressive therapy have been described in several longitudinal cohorts, where PLWH with apparently successful treatment outcomes continue to exhibit markers of immune activation despite adequate CD4+ recovery [27].

Large prospective studies have further shown that persistently low CD4/CD8 ratios are associated with increased risks of both AIDS-related and non-AIDS morbidity, including cardiovascular disease, malignancies, and mortality. These observations support the concept that the CD4/CD8 ratio captures aspects of immune recovery not fully reflected by CD4+ counts alone [33]. Complementary population-level analyses have also demonstrated that incorporating the CD4/CD8 ratio into routine monitoring may help identify individuals who remain at increased risk of long-term complications despite effective viral suppression and apparently adequate CD4+ recovery [41]. In this context, the persistence of ratio inversion observed in our cohort suggests that a considerable proportion of long-term treated individuals may continue to experience underlying immune dysregulation that is not evident when evaluating CD4+ counts alone [13].

Another factor that may contribute to persistent ratio inversion is premature immunological aging, or immunosenescence [42]. Chronic HIV infection, even under effective antiretroviral therapy, has been associated with accelerated immune aging characterized by expansion of terminally differentiated CD8+ T cells, contraction of naïve T-cell pools, and increased expression of senescence markers such as CD57 and KLRG1. These changes may impair immune homeostasis and limit the capacity for complete immune reconstitution despite prolonged viral suppression [11,43]. Persistent expansion of senescent or exhausted T-cell populations has been linked to chronic inflammation, reduced immune responsiveness, and increased susceptibility to age-related comorbidities among people living with HIV [44]. Within this framework, the CD4/CD8 ratio has been proposed as a simple surrogate marker of senescence-associated immune imbalance, as ratio inversion reflects both incomplete CD4+ restoration and accumulation of differentiated CD8+ T-cell populations [45].

In the Romanian HIV cohort, characterized by prolonged infection duration and extensive treatment exposure, persistent CD4/CD8 ratio inversion across treatment response groups may therefore reflect long-standing immune remodeling associated with chronic antigenic stimulation. Individuals infected during childhood have experienced decades of immune activation and viral antigen exposure, factors that may accelerate immunosenescence and contribute to sustained CD8+ expansion observed in the present study [46,47].

Another relevant observation is the absence of correlation between the CD4/CD8 ratio and temporal variables such as duration of HIV infection or antiretroviral therapy. This suggests that ratio normalization does not progressively occur simply as a consequence of longer treatment exposure. Instead, the ratio may reach a plateau after viral suppression is achieved, reflecting structural alterations in T-cell homeostasis that are not easily reversed. Mechanisms such as impaired thymic output, altered lymphocyte differentiation, and persistent inflammatory signaling may limit full immune restoration even during long-term suppressive therapy [7].

The regression analyses examining determinants of discordant treatment responses revealed limited explanatory power of conventional clinical variables. Male sex was the only factor significantly associated with both immunological and virological discordance. Sex-related differences in immune responses have been described in multiple HIV cohorts, with women generally demonstrating stronger antiviral responses and more favorable immune recovery trajectories [27].

The low explanatory power of the multinomial model suggests that discordant immune responses are likely influenced by factors not captured in routine clinical datasets. Potential determinants include treatment adherence, emergence of drug resistance mutations, viral replication in anatomical reservoirs, and persistent inflammatory signaling. Increasing attention has also focused on chronic antigenic stimulation from latent viral reservoirs and coinfections as contributors to sustained immune activation during suppressive therapy [36,48].

Residual immune activation during treated HIV infection likely results from multiple interacting mechanisms, including LLV transcription from persistent reservoirs, microbial translocation, and chronic stimulation by coinfections such as cytomegalovirus. Together, these processes maintain inflammatory signaling pathways that may contribute to heterogeneous patterns of immune reconstitution despite effective viral suppression [49,50].

The evaluation of viral hepatitis coinfections revealed no significant association between HBV or HCV status and discordant treatment responses. Although coinfections may contribute to systemic inflammation, their influence on immune recovery during antiretroviral therapy appears to vary across populations and clinical contexts [27].

Finally, the analysis of opportunistic infection burden showed that current treatment response classification was not independently associated with the presence or number of historical opportunistic infections. Instead, markers of historical disease severity, particularly CDC stage and duration of HIV infection, were the main predictors. Interestingly, duration of HIV infection showed an inverse association with opportunistic infection history. Although this finding may appear counter-intuitive, it likely reflects a survivor bias effect, whereby individuals with longer infection duration represent a selected subgroup of long-term survivors who may have experienced fewer severe opportunistic events or who had more favorable clinical trajectories. This effect may be particularly relevant in the Romanian cohort, which includes individuals with prolonged survival following early-life infection. In addition, the cumulative history of opportunistic infections reflects past episodes of immunosuppression rather than current immune status and may therefore not directly correlate with longer duration of treated infection [1,33].

Several strengths of this study deserve emphasis. The analysis included a relatively large cohort with prolonged antiretroviral exposure and detailed clinical and immunological characterization. The integration of multiple analytical approaches, including correlation analyses, multinomial regression, and count-based regression models, allowed a comprehensive evaluation of immune dysregulation patterns across treatment response groups. In addition, the Romanian HIV cohort provides a unique setting for investigating long-term immune reconstitution because of its prolonged infection duration and complex treatment histories.

Nonetheless, several limitations should be acknowledged. The cross-sectional design does not allow assessment of longitudinal trajectories of CD4+, CD8+, or CD4/CD8 ratio changes over time. In addition, the classification of virological discordance was based on a single viral load measurement, which may not fully capture longitudinal virological dynamics and may lead to potential misclassification of transient viral blips as persistent viremia. Moreover, the virological discordance group included individuals with both low-level viremia and virological failure, which represent biologically and clinically distinct conditions. The absence of drug resistance data and longitudinal viral load follow-up limited further stratification of this group and precluded differentiation between transient viremia and resistance-driven virological failure. In addition, the wide range of plasma viral load values within this group reflects substantial heterogeneity in virological control, which may have influenced the interpretation of virological discordance. Also, several potentially relevant biological determinants were not available, including cytomegalovirus serostatus, markers of microbial translocation, immune activation markers such as CD38 and HLA-DR expression, and measures of treatment adherence. Furthermore, detailed characterization of viral hepatitis coinfections was limited by the retrospective nature of the data, as information regarding active versus resolved infection, antiviral treatment status, the presence of triple infection (HIV–HBV–HCV), and hepatitis D virus coinfection was not consistently available, which may have influenced the assessment of their impact on immune and virological outcomes. These factors have been implicated in persistent immune dysregulation and may contribute to discordant responses during suppressive therapy.

Future studies incorporating longitudinal immune monitoring and advanced immunophenotyping, including analysis of T-cell differentiation states, exhaustion markers, and pathogen-specific immune responses, may further clarify the mechanisms underlying persistent immune dysregulation during treated HIV infection and help identify strategies aimed at restoring immune balance in individuals with discordant immune–virologic responses.

## 5. Conclusions

In this cohort of 462 PLWH receiving long-term antiretroviral therapy, discordant immune–virologic responses remained common, affecting, together, nearly one-third of individuals. Despite high rates of viral suppression, some of them continued to exhibit incomplete CD4+ T-cell recovery or detectable viremia. Persistent CD8+ T-cell expansion was observed across all treatment response clinical classifications, suggesting that chronic CD8+ elevation represents a shared immunological background in long-term treated HIV infection rather than a characteristic specific to a given clinical classification.

Within this context, the degree of CD4+ T-cell recovery appears to be the principal determinant of variation in the CD4/CD8 ratio. Our findings indicate that CD4/CD8 ratio inversion reflects not only incomplete CD4+ restoration but also sustained CD8+-driven immune activation and immunological aging. The CD4/CD8 ratio therefore provides integrative information on immune balance beyond absolute CD4+ counts alone and may help identify individuals who remain at risk of persistent immune dysfunction despite effective viral suppression.

Conventional clinical variables explained only a limited proportion of variability in discordant immune–virologic response groups, suggesting that additional biological determinants, like persistent immune activation, viral reservoir dynamics, or host inflammatory pathways, may contribute to heterogeneous immune recovery during long-term therapy. These findings reinforce the concept that virological suppression alone does not fully capture the complexity of immune restoration in treated HIV infection and support the use of biomarker-informed monitoring strategies to better characterize immune dysregulation in chronic HIV disease.

## Figures and Tables

**Table 1:** Demographic, clinical, immunological and virological characteristics of the study cohort according to treatment response clinical classification.

Variable	Total (N = 462)	CR (n = 320)	ID (n = 67)	VD (n = 75)	*p*-Value
Demographics					
Age (years)	40.0 [35.0–50.0]	39.0 [34.0–50.2]	37.0 [34.0–49.5]	44.0 [37.0–51.0]	0.071
Sex, male	286 (61.9%)	187 (58.4%)	48 (71.6%)	51 (68.0%)	0.064
Environment, urban	345 (74.7%)	241 (75.3%)	50 (74.6%)	54 (72.0%)	0.838
Disease history					
Duration HIV (years)	18.0 [11.0–25.0]	18.0 [11.0–25.0]	17.0 [11.0–26.0]	21.0 [12.0–24.0]	0.927
Duration ART (years)	14.0 [9.0–21.8]	13.0 [9.0–21.0]	15.0 [10.0–23.0]	16.0 [11.0–21.0]	0.182
No. ARV regimens	5.0 [2.0–12.0]	5.0 [2.0–12.0]	5.0 [1.5–12.0]	6.0 [3.0–11.0]	0.630
CD4 nadir (cells/µL)	163 [53–314]	163 [57–314]	175 [45–335]	155 [44–309]	0.740
CDC Clinical Stage					
Stage A	58 (12.6%)	49 (15.3%)	4 (6.0%)	5 (6.7%)	0.045
Stage B	114 (24.7%)	70 (21.9%)	20 (29.9%)	24 (32.0%)	
Stage C	290 (62.8%)	201 (62.8%)	43 (64.2%)	46 (61.3%)	
Immunological Parameters					
CD4+ count (cells/µL)	532.5 [309–765]	638.5 [462–820]	174 [157–191]	470 [334–655]	<0.001
CD8+ count (cells/µL)	788 [565–1067]	790.5 [570–1047]	755 [524–1048]	792 [568–1207]	0.571
CD4/CD8 ratio	0.6 [0.4–1.1]	0.8 [0.5–1.2]	0.2 [0.2–0.3]	0.6 [0.4–0.8]	<0.001
CD4/CD8 Ratio Inversion					
Ratio inversion (<1.0)	329 (71.2%)	202 (63.1%)	67 (100%)	60 (80.0%)	<0.001
Severe inversion (≤0.5)	185 (40.0%)	89 (27.8%)	63 (94.0%)	33 (44.0%)	
Virological Status					
Viral suppression (<50 copies/mL)	387 (83.8%)	320 (100.0%)	67 (100.0%)	0 (0.0%)	<0.001
LLV (50–199 copies/mL)	36 (7.7%)	0 (0.0%)	0 (0.0%)	36 (48.0%)	
VF (200–999 copies/mL)	10 (2.2%)	0 (0.0%)	0 (0.0%)	10 (13.3%)	
VF (≥1000 copies/mL)	29 (6.3%)	0 (0.0%)	0 (0.0%)	29 (38.7%)	
Viral Hepatitis Coinfections					
HCV coinfection	172 (37.2%)	124 (38.8%)	27 (40.3%)	21 (28.0%)	0.190
HBV coinfection	190 (41.1%)	130 (40.6%)	29 (43.3%)	31 (41.3%)	0.922
Opportunistic Infections					
Any OI history	236 (51.1%)	165 (51.6%)	33 (49.3%)	38 (50.7%)	0.940
Number of OIs	1.0 [0.0–2.0]	1.0 [0.0–2.0]	1.0 [0.0–2.0]	1.0 [0.0–2.0]	0.761
Tuberculosis	56 (12.1%)	38 (11.9%)	7 (10.4%)	11 (14.7%)	0.722
Pneumocystis pneumonia	24 (5.2%)	18 (5.6%)	0 (0.0%)	6 (8.0%)	0.083
Toxoplasmosis	42 (9.1%)	32 (10.0%)	4 (6.0%)	6 (8.0%)	0.544
Cytomegalovirus	143 (31.0%)	101 (31.6%)	25 (37.3%)	17 (22.7%)	0.155
EBV	58 (12.6%)	43 (13.4%)	7 (10.4%)	8 (10.7%)	0.690
Herpes simplex	79 (17.1%)	53 (16.6%)	13 (19.4%)	13 (17.3%)	0.853
Mycoplasma pneumoniae	44 (9.5%)	32 (10.0%)	5 (7.5%)	7 (9.3%)	0.812
Candidiasis	55 (11.9%)	35 (10.9%)	7 (10.4%)	13 (17.3%)	0.282
Kaposi sarcoma	5 (1.1%)	4 (1.2%)	0 (0.0%)	1 (1.3%)	0.650
Salmonella	39 (8.4%)	24 (7.5%)	9 (13.4%)	6 (8.0%)	0.283

Continuous variables are presented as median [IQR], and categorical variables as n (%). *p*-values were calculated using the Kruskal–Wallis test for continuous variables and the Pearson χ^2^ test for categorical variables. The virological discordant (VD) group includes individuals with both low-level viremia and virological failure, reflecting heterogeneity in virological control. Viral load distribution statistics are reported only for the VD group. CR, concordant response; ID, immunological discordance; VD, virological discordance; ART, antiretroviral therapy; LLV, low-level viremia; VF, virological failure; OI, opportunistic infection; EBV, Epstein–Barr virus.

**Table 2:** Detailed distribution of virological status within the virological discordant group.

Parameters	LLV (50–199 Copies/mL) (n = 36)	VF (200–999 Copies/mL) (n = 10)	VF (≥1000 Copies/mL) (n = 29)
Viral load (copies/mL)			
Median [IQR]	79 [69–101]	438 [293–526]	36,400 [3280–143,000]
Min–max	54–174	211–624	1090–1,020,000

LLV, low-level viremia; VF, virological failure; IQR, interquartile range.

**Table 3:** Immune dysregulation across treatment response group stratification.

Variable	CR (n = 320)	ID (n = 67)	VD (n = 75)	*p*-Value	ES
CD4/CD8 ratio	0.8 [0.5–1.2]	0.2 [0.2–0.3]	0.6 [0.4–0.8]	<0.001	ε^2^ = 0.25
CD8+ count	790.5 [570–1047]	755 [524–1048]	792 [568–1207]	0.571	ε^2^ = 0.002
CD4+ count	638.5 [462–820]	174 [157–191]	470 [334–655]	<0.001	ε^2^ = 0.33
CD4/CD8 < 1.0, n (%)	202 (63.1%)	67 (100%)	60 (80.0%)		V = 0.295
CD4/CD8 ≤ 0.5, n (%)	89 (27.8%)	63 (94.0%)	33 (44.0%)		V = 0.469

Effect size (ES) is reported as ε^2^ for Kruskal–Wallis tests and Cramér’s V for χ^2^ tests. Post hoc pairwise (Bonferroni): all pairs significant for CD4/CD8 ratio and CD4 count. CD8 count did not differ across group stratification.

**Table 4:** Multinomial logistic regression: determinants of ID and VD vs. CR. RRR = relative risk ratio.

Predictor	RRR	95% CI	*p*-Value	RRR	95% CI	*p*-Value
	ID vs. CR			VD vs. CR		
Age (years)	0.985	0.960–1.011	0.256	1.010	0.988–1.033	0.375
Sex (Male)	2.160	1.177–3.963	0.013 *	1.789	1.016–3.150	0.044 *
Duration ART	1.054	0.991–1.120	0.093	1.054	0.994–1.117	0.078
CD4+ Nadir	1.000	0.998–1.001	0.838	0.999	0.998–1.001	0.233
HCV	1.030	0.596–1.781	0.914	0.608	0.347–1.065	0.082
HBV	0.999	0.576–1.733	0.998	0.977	0.576–1.660	0.933
No. ARV regimens	0.974	0.927–1.023	0.295	0.978	0.933–1.024	0.336
CDC Stage	1.229	0.773–1.953	0.383	1.032	0.669–1.592	0.886

Reference category: CR. RRR, relative risk ratio. Model likelihood ratio χ^2^ = 20.78 (*p* = 0.187); pseudo-R^2^ = 0.027. Duration of cART was included in the model due to collinearity with duration of HIV infection (Spearman ρ = 0.809). *, *p* < 0.05.

**Table 5:** Binary logistic regression: predictors of severe CD4/CD8 ratio inversion (≤0.5). aOR = adjusted odds ratio.

Predictor	aOR	95% CI	*p*-Value
Age (years)	0.982	0.962–1.002	0.075
Sex (Male)	1.242	0.778–1.981	0.364
Duration ART (years)	0.981	0.942–1.021	0.341
CD4+ Nadir	1.000	0.999–1.002	0.424
HCV Coinfection	0.948	0.605–1.486	0.817
HBV Coinfection	0.673	0.430–1.056	0.085
CDC Stage	1.092	0.772–1.545	0.617
ID (vs. CR)	43.491	15.172–124.674	<0.001
VD (vs. CR)	2.179	1.277–3.718	0.004

aOR, adjusted odds ratio; CI, confidence interval. Model performance: McFadden pseudo-R^2^ = 0.194; AUC = 0.751; AIC = 521.1.

**Table 6:** Spearman correlations between CD4/CD8 ratio and clinical variables with FDR correction.

Correlation Pair	ρ (Spearman)	*p*-Value	*p*-Value (FDR)
CD4/CD8 vs. CD8+ count	−0.563	<0.001	<0.001
CD4/CD8 vs. CD4+ count	0.791		
CD4/CD8 vs. CD4+ Nadir	−0.069	0.138	0.247
CD4/CD8 vs. age	0.065	0.165	0.247
CD4/CD8 vs. duration of HIV	0.000	0.999	0.999
CD4/CD8 vs. duration of cART	−0.015	0.754	0.904

FDR-adjusted *p*-values were calculated using the Benjamini–Hochberg correction.

**Table 7:** Viral hepatitis coinfections across treatment response groups.

Variable	CR (n = 320)	ID (n = 67)	VD (n = 75)	*p*-Value	ES
HCV coinfection, n (%)	124 (38.8%)	27 (40.3%)	21 (28.0%)	0.190	V = 0.085
HBV coinfection, n (%)	130 (40.6%)	29 (43.3%)	31 (41.3%)	0.922	V = 0.019

**Table 8:** Opportunistic infection models. Left: binary logistic (aOR). Right: negative binomial (IRR). Both include duration of HIV infection.

Predictor	aOR	95% CI	*p*-Value	IRR	95% CI	*p*-Value
	Binary Logistic	Any OI		Neg. Binomial	No. of OIs	
CD4+ Nadir	0.999	0.998–1.001	0.323	1.000	0.999–1.000	0.244
CDC Stage	1.299	0.959–1.760	0.091	1.192	1.010–1.406	0.037
Duration HIV (years)	0.964	0.939–0.990	0.007 *	0.977	0.963–0.990	<0.001
Age (years)	1.012	0.996–1.029	0.148	1.000	0.991–1.009	0.978
Sex (Male)	0.952	0.637–1.423	0.810	1.053	0.847–1.310	0.641
HCV Coinfection	1.056	0.717–1.555	0.783	1.066	0.869–1.307	0.541
HBV Coinfection	0.968	0.659–1.424	0.870	1.004	0.818–1.233	0.971
ID (vs. CR)	0.906	0.529–1.552	0.720	0.950	0.712–1.267	0.726
VD (vs. CR)	0.918	0.550–1.534	0.745	0.980	0.745–1.290	0.887

Binary logistic regression was used to evaluate predictors of any OI history, and negative binomial regression to evaluate predictors of the number of OIs. Both models included identical predictor sets. Overdispersion in the count model justified the use of negative binomial regression (variance 1.58 > mean 1.18). aOR, adjusted odds ratio; IRR, incidence rate ratio; CI, confidence interval; *, *p* <0.05.

## Data Availability

The raw data supporting the conclusions of this article will be made available by the first author on request.

## Abbreviations

The following abbreviations are used in this manuscript:

aOR	Adjusted odds ratio
ART	Antiretroviral therapy
CDC	Centers for Disease Control and Prevention
CI	Confidence interval
cART	Combination antiretroviral therapy
CD4+	Cluster of differentiation 4 positive T lymphocytes (CD4+ T cells)
CD8+	Cluster of differentiation 8 positive T lymphocytes (CD8+ T cells)
CR	Concordant response
EBV	Epstein–Barr virus
FDR	False discovery rate
HBV	Hepatitis B virus
HCV	Hepatitis C virus
HIV	Human immunodeficiency virus
HIV-1	Human immunodeficiency virus type 1
HLA-DR	Human leukocyte antigen-dr isotype
ID	Immunological discordance
IRR	Incidence rate ratio
IQR	Interquartile range
KLRG1	Killer cell lectin-like receptor subfamily G member 1
LLV	Low-level viremia
OI	Opportunistic infection
PLWH	People living with HIV
PD-1	Programmed cell death protein 1
RRR	Relative risk ratio
ROC	Receiver operating characteristic
VF	Virological failure
VD	Virological discordance
